# Effects of high-frequency nanosecond pulses on prostate cancer cells

**DOI:** 10.1038/s41598-021-95180-7

**Published:** 2021-08-04

**Authors:** Aleksander Kiełbik, Wojciech Szlasa, Vitalij Novickij, Anna Szewczyk, Magdalena Maciejewska, Jolanta Saczko, Julita Kulbacka

**Affiliations:** 1 Medical University Hospital, Borowska 213, 50-556 Wrocław, Poland; 2 Department of Molecular and Cellular Biology, Faculty of Pharmacy, Wroclaw Medical University, Wrocław, Poland; 3 Faculty of Medicine, Wroclaw Medical University, Wrocław, Poland; 4 Institute of High Magnetic Fields, Vilnius Gediminas Technical University, Vilnius, Lithuania; 5 Department of Animal Developmental Biology, Institute of Experimental Biology, University of Wroclaw, 50-328 Wrocław, Poland; 6 Hirszfeld Institute of Immunology and Experimental Therapy, Polish Academy of Sciences, 53-114 Wrocław, Poland

**Keywords:** Prostate cancer, Cancer therapy

## Abstract

Electroporation with pulsed electric fields show a potential to be applied as an experimental focal therapy of tumors. Sub-microsecond regime of electric pulses displays unique electrophysical features operative in cells and membranes. Recently, MHz compression of nanosecond pulses electric fields (nsPEFs) bursts proved to enhance the effectiveness of the therapy. High morbidity of prostate cancer (PCa) and risk of overtreatment associated with this malignancy call for new minimal-invasive treatment alternative. Herein we present the in vitro study for developing applications based on this new technology. In this study, we used flow cytometric analysis, cell viability assay, caspase activity analysis, wound healing assay, confocal microscopy study, and immunofluorescence to investigate the biological effect of high-frequency nsPEFs on PCa cells. Our results show that high-frequency nsPEFs induces the permeabilization and cell death of PCa cells. The cytotoxicity is significantly enhanced in MHz compression of pulses and with the presence of extracellular Ca^2+^. High-frequency nsPEFs trigger changes in PCa cells’ cytoskeleton and their mobility. The presented data show a therapeutic potential of high-frequency nsPEFs in a PCa setting. The sub-microsecond regime of pulses can potentially be applied in nanosecond electroporation protocols for PCa treatment.

## Introduction

Intense pulsed electric fields (PEFs) can be applied to permeabilize biomembranes^1^. This phenomenon is known as electroporation and found its use among others in the minimal-invasive treatment of different types of cancer. Clinically, commonly series of microsecond pulses are applied in various approaches such as electrochemotherapy or irreversible electroporation. Schoenbach and Beebe (2001) presented that biomembranes can also be permeabilized with 60 ns pulses and initiated a series of studies investigating the effect of sub-microsecond PEFs^2^.

Initially, it was observed that nanosecond pulsed electric fields (nsPEFs) preferably affect cells interior acting on intracellular biomembranes^3^. Indeed, ns electroporation induces the dissipation of mitochondria membrane potential what eventually leads to cell death^4,5^. The permeabilization of other intracellular compartments such as the endoplasmic reticulum triggers the release of calcium and apoptosis of the cells^6^. Apart from intracellular compartments, multiple studies confirm that high-voltage submicrosecond PEFs can permeabilize plasma membrane^7,8^. The nsPEFs, similar to longer microsecond PEFs, trigger permeabilization increasing the transmembrane voltage^9^. However, comparing to standard microsecond PEFs, the size of permeable spots is smaller, so they can not always be detected by conventional dyes^10^. The permeabilization induced by ns pulses is a long-lived process that enables small molecules uptake for a longer time^11^. Consequently, the permeabilization is followed by ions inflow^12^, cell swelling^13^, cytoskeleton destabilization^11^ and eventually necrotic or apoptotic cell death^14^.

In vivo studies presented that nsPEFs trigger apoptotic cell death and result in the restriction of tumor vascularization^15,16^. Moreover, research on the animal models show signs of the systemic anti-cancer immune response^17,18^.

Prostate cancer (PCa) remains the second most often diagnosed malignancy among men worldwide^19^. A systematic review of autopsy studies reported a prevalence ranging from 48 to 71% by patients over 79 years^20^. However, in most cases, PCa is associated with long life expectancy, and the radical therapy of low and intermediate-risk PCa is often unnecessary. Therefore, few focal therapies were developed to prevent overtreatment and provide patients with the alternative to radical prostatectomy. Among others, electroporation constitutes a new promising treatment modality for PCa^21^. Differently from commonly used microsecond pulses, we propose the application of pulses in the nanosecond range.

The major limitation for sub-microsecond pulses is the need for high-voltage bursts to induce the cellular response. Therefore, usually pulses up over 10 kV/cm are applied. Novickij et al. (2018) presented that to overcome this limitation short pulses can be delivered in higher frequencies^22^. Namely, pulses should be applied in intervals shorter than the relaxation time of induced transmembrane potential. MHz monopolar nsPEFs remained poorly investigated due to the technological challenges of generating high voltage monopolar pulses in the MHz range^22^. Recent research proved that MHz ns pulses could be applied for gene delivery^23^ and nerve excitation^24^.

Primary, we determined the correlation between the frequency of pulses and permeabilization of cells. Secondly, we evaluated the cytotoxic effect of conventional and high-frequency nsPEFs in the buffer with and without a high concentration of extracellular Ca^2+^. The Ca^2+^ uptake dynamic after bursts with different frequency PEFs was determined by a fluorescence microscopy study. The biological effects of MHz nsPEFs remain poorly investigated. Therefore, our experiments aimed at defining the effect of high-frequency bursts on the cytoskeleton and mobility of the cells.

## Results

### The permeabilization rate of PCa cells depends on the electric field intensity and frequency of PEFs

**Figure 1:**
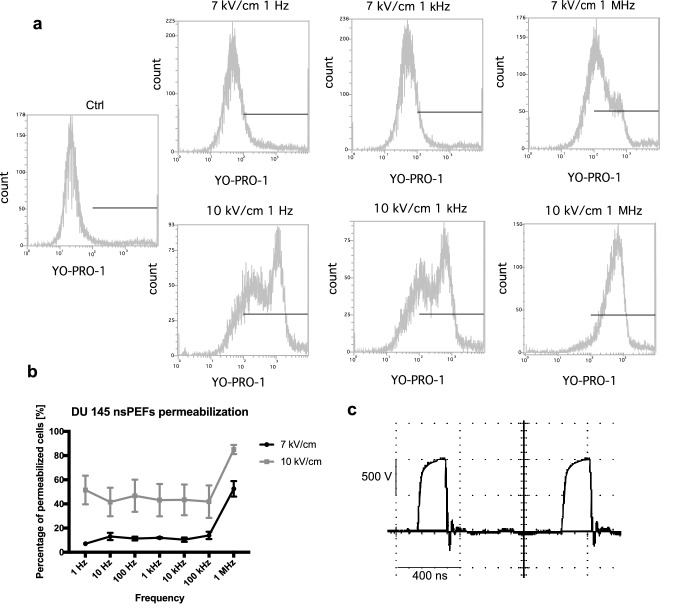
Permeabilization of DU 145 cells by 25, 200 ns PEFs bursts evidenced by YO-PRO-1 dye uptake. Cells were permeabilized in suspension in a 1-mm gap electroporation cuvette. (**a**) Flow cytometry analysis data presented on one-dimensional histograms. The graphs represent the data form one representative experiment. (**b**) The permeability of cells as a function of electric field frequency. The graph is representative of three independent experiments. Data are mean ± SD (n = 3 independent experiments). The effect of nsPEF was measured at two different electric field intensities. 25-pulse, 200-ns bursts become significantly more efficient at MHz. (**c**) The shape of high-frequency nsPEF delivered to 1 mm electroporation cuvettes.

Initially, PEFs parameters were optimized to achieve a high rate of responding cells. Figure 1 presents the effect of the standalone series of 200 ns pulses in different frequencies on the DU 145 cells permeabilization. The permeabilization depends on the electric field intensity and frequency of PEFs. Pulses delivered at MHz are significantly more efficient in electroporating cells. 25, 7 kV/cm PEFs in MHz frequency result in comparable permeabilization to 25, 10 kV/cm PEFs in lower frequencies (Fig. 1b).

### The antitumor effect of PEFs is potentiated by the increasing number of pulses in MHz frequency and administration of Ca^2+^

**Figure 2:**
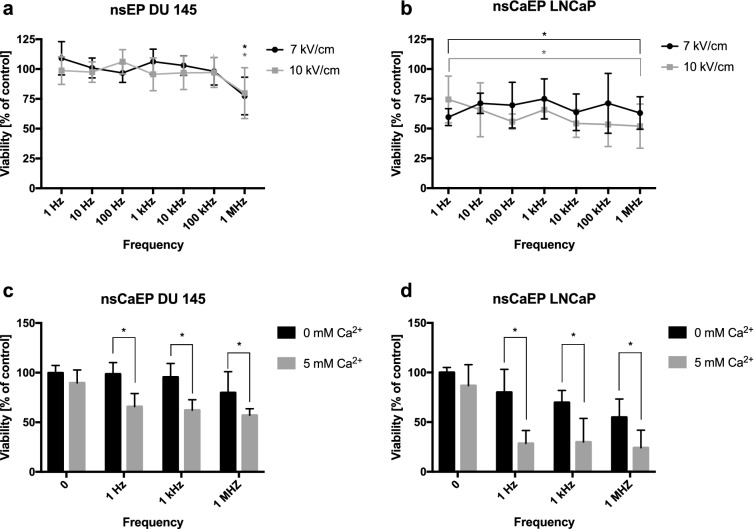
Viability of DU 145 and LNCaP cells after expousure to 25, 200 ns PEFs (**a**–**d**). Cells were permeabilized in suspension in a 1-mm gap electroporation cuvette. Viability of the cells as a function of electric field frequency. (**a**, **b**) The effect of PEFs was measured at two different electric field intensities. (**c**, **d**) Viability of cells after exposure to PEFs in medium with different Ca^2+^ concentration. Viability was assessed with MTT assay. Graphs are representative of at least 3 independent experiments. Data are mean ± SD (n = 3–5 independent experiments). (*) indicates statistically significant differences between the sample and control (**a**, **b**), or pair of samples (**c**, **d**) (ANOVA, *p* < 0.05).

We evaluated the impact of PEFs on the PCa cells’ viability. The series of 25, 200 ns pulses has little impact on the DU 145 cells survival (Fig. 2a). A significant decrease is observed only when pulses in MHz frequency are applied. The effect of PEFs is more evident on the LNCaP cells (Fig. 2b). However, the LNCaP cells lethality is independent of PEFs frequency. A profound increase in cell death is achieved after exposure to PEFs in a buffer containing Ca^2+^. High Ca^2+^ concentration facilitates the cytotoxicity of 25, 200 ns PEFs on both cell lines in all investigated frequencies (Fig. 2c, d).

**Figure 3:**
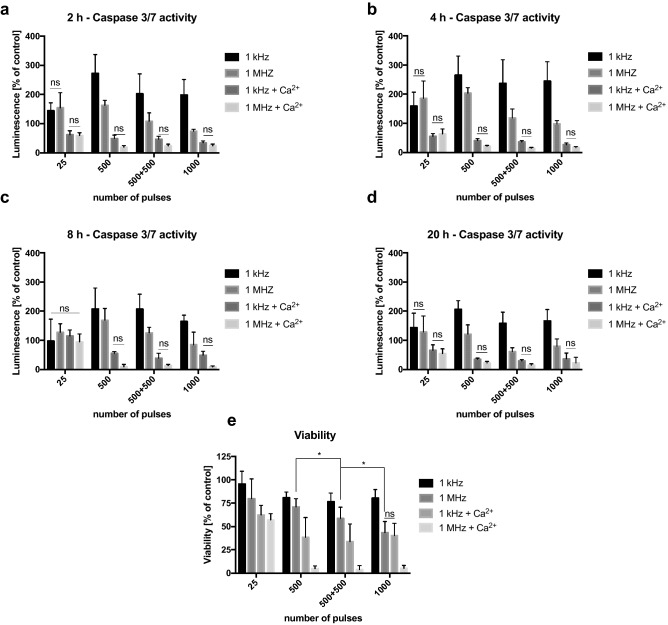
Viability and Caspase 3/7 activity of the DU 145 cells after high-frequency PEFs application. Cells were exposed in suspension in a 1-mm gap electroporation cuvette (200 ns duration, 10 kV). (**e**) The effect of pulse number in two different frequencies and different extracellular calcium concentration. The viability was assessed with MTT assay 24 h after PEFs expousure. (**a**–**d**) The Caspase 3/7 activity in the cells after exposure to a different number of PEFs in two frequencies and different extracellular Ca^2+^ concentration. The caspase 3/7 activity was determined with Caspase-Glo 3/7 Assay from Promega 2, 4, 8 and 20 h after elctroporation. Graphs are representative of at least 3 independent experiments. Data are mean ± SD (n = 3–4 independent experiments). (ns) indicates no statistically significant difference between the pair of samples (*) indicates statistically significant differences between the pair of samples (ANOVA, *p* < 0.05).

Bursts with a higher number of 200 ns PEFs increase the cytotoxicity of the therapy. Figure 3e presents the correlation between the number of applied pulses and cancer cell viability. Results suggest that if pulses are delivered in MHz frequency, their increasing number significantly affects cell viability. Moreover, the effect is more pronounced when MHz PEFs are delivered in one burst, not intervals. Differently, kHz compression of 200 ns PEFs shows no significant difference in cytotoxicity between PEFs in one or two bursts. The cytotoxic effect of a higher numer of 200 ns pulses was potentiated by the addition of Ca^2+^ to the electroporation medium (Fig. 3e). In those probes, MHz compression of PEFs showed significant superiority to kHz PEFs and very high efficiency in decreasing PCa cells viability.

Figures 3a–d show apoptotic death markers activity after delivery of different 200 ns high-frequency ns bursts. Caspase 3 and 7 activity was studied to validate whether high-frequency nsPEFs trigger apoptosis. The time courses of caspases activity indicate the highest expression of caspase 3 and 7 4 h after the exposure to PEFs.The cells subjected to PEFs with Ca^2+^, show low expression of apoptotic death markers after the therapy. After permeabilization without Ca^2+^ in EP buffer cells enter the apoptotic pathways, partially contributing to the observed decrease of cell viability after 24 h. Interestingly, the expression of apoptotic markers is significantly higher after bursts in kHz frequency comparing to MHz.

### Ca^2+^ transients dynamic evoked by ns bursts depends on the frequency of PEFs

**Figure 4:**
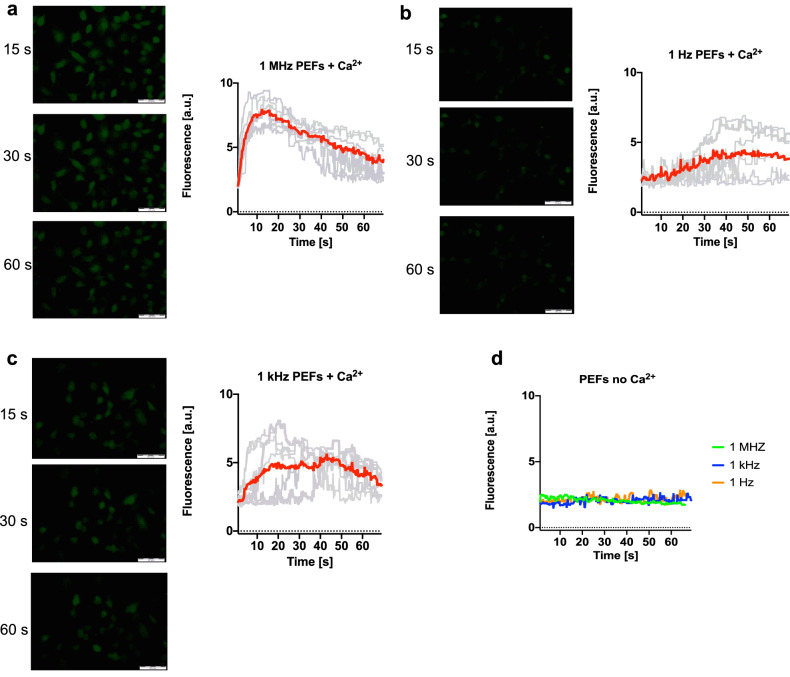
Ca^2+^ transients dynamic evoked by 200 ns bursts. The adherent cells were permeabilized with two needle electrode with bursts of 25, 5 kV/cm, 200 ns PEFs in different frequencies. The dynamic of Ca^2+^ uptake after nanosecond permeabilization was presented as traces of response in selected individual cells (grey lines, 7 cells per plot) and their average (colour lines). The cells were stained with Fluo-8 dye. Pulses were delivered at the beginning of the observation and the cells were observed for 60 s. Images were analyzed by ImageJ software (Version: 2.1.0/1.53C; https://imagej.net/Fiji)^28^.

Figure 4 shows visible differences in Ca^2+^ uptake and efflux dynamic after bursts with 25, 200 ns PEFs in different frequencies. Cells exposed to PEFs without extracellular Ca^2+^ do not respond with a detectable Ca^2+^ rise. The shapes of the response of cells subjected to PEFs with Ca^2+^ vary depending on the frequency. The fluorescence rise and fall time are notably different. Bursts of MHz pulses resulted in the sharp rise of intracellular Ca^2+^ with subsequent smooth and fast (60 s) return to the resting level (Fig. 4a). After the exposure to pulses in kHz and Hz frequency, Ca^2+^ transients do not occur immediately after bursts. Moreover, the increase of intracellular Ca^2+^ level is lower than MHz pulses (Fig. 4b, c).

### High-frequency nsPEFs trigger changes in PCa cells’ cytoskeleton and their mobility

**Figure 5:**
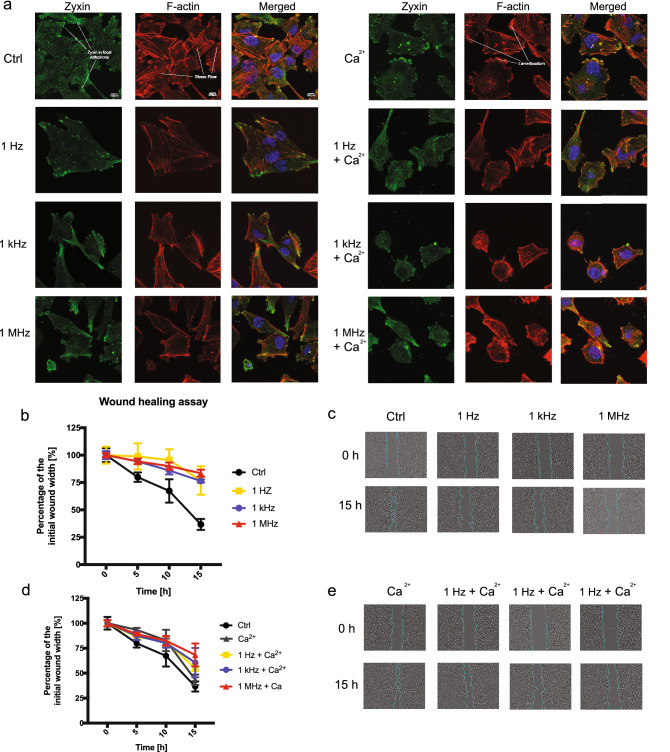
PCa cells morphology and mobility after exposure to 25, 10 kV/cm, 200 ns pulses in different frequencies with and without extracellular Ca^2+^. (**a**) The representative photographs of cells exposed to bursts of nsPEFs. Confocal laser scanning microscopy visualize the rearrangement of zyxin (green fluorescent dye), F-actin (red fluorescent dye) nucleus (blue florescent dye) structure in DU 145 cells. (**b**, **c**) The percentage of a healed wound as a function of time. Images were analyzed by ImageJ software (Version: 2.1.0/1.53C; https://imagej.net/Fiji)^28^. (**d**, **e**) Images of wound gradually invaded by migrating cells. Images were taken in a given time interval. The graph represents the data from the three replicates of an individual experiment. Data are mean ± SD (n = 3 replicates).

Fluorescence staining after exposure to 25, 200 ns PEFs revealed changes in the cell morphology (Fig. 5a). High-frequency PEFs trigger the disruption of lamellipodia and stress fibers. F-actin after high-frequency ns bursts accumulates in peripheral parts of cells. This effect is less evident after exposure to PEFs in 1 Hz frequency. High-frequency PEFs in buffer containing Ca^2+^ ions trigger prominent changes in cell morphology. The latter results in cell rounding and the creation of honeycomb-like structures. In untouched cells, zyxin localizes with actin fibers on the membrane protrusions. Figure 5a shows that standalone bursts of 25, 200 ns high-frequency PEFs have a mild effect on zyxin localization. However, the changes after permeabilization with Ca^2+^ are remarkable and indicate zyxin translocalization from focal adhesions. Cells after PEFs application present impaired mobility. Figure 5b–e reports the results of the wound healing assays. The cells were scrutinized for 15 h until the colonies of the control group connected. This assay shows a slight difference between probes permeabilized with and without Ca^2+^ ions.

## Discussion

Our in vitro study shows the potential of high-frequency nsPEFs for prostate cancer treatment. Bursts of 25, 200 ns pulses result in a high permeability rate of the cells. Nevertheless, the latter is not reflected by their high mortality. However, note that cells after treatment were kept in the optimal condition in a growth medium containing serum what might significantly increase their viability after PEFs application^30,31^. For consistency, we also provide the data for LNCaP cells, but most experiments were performed on DU 145 cells. LNCaP cells do not produce a uniform monolayer but grow in clusters. Moreover, they attach only lightly to the substrate. Consequently, we choose DU 145 cell line as more appropriate for the study. In our experiments, two PCa cell lines show different sensitivity to ns bursts. The cytotoxic effect of nsPEFs varies across different cell lines with no visible dependence on cell size and other morphology features^32^.

Herein we studied two approaches to enhance the effectiveness of high-frequency nsPEF. Namely, we increased the number of pulses and applied calcium ions. The increase of high-frequency PEFs number significantly upregulates the cytotoxic effect of the therapy. In our study, the latter is more pronounced when pulses in MHz frequencies are delivered. We also detected the favorable effect of unfractionated MHz PEFs. The pause between the series of pulses can result in the sensitization of cells, which increases the cytotoxicity of fractionated therapy^33^. The effect of cell sensitization was not observed in our experiment. The result can be explained by the fact that the cells were electroporated in cuvettes. The applied method does not prevent the cells’ sedimentation and their random rotation, affecting the sensitization^33^. Recently we proved that calcium ions could enhance the cytotoxicity of microsecond electroporation of prostate adenocarcinoma cells^34^. This study confirms that a burst of high-frequency nsPEFs with the presence of the extracellular Ca^2+^ also triggers extensive cell death. After 24 h the population of cells permeabilized with Ca^2+^ was significantly reduced. We observed higher activity of casp-3/7 8 h after standalone PEFs delivery. Moreover, an increase in apoptotic markers after kHz PEFs over MHz PEFs was detected. We hypothesize that MHz and permeabilization with extracellular Ca^2+^ pulses could result in extensive non-apoptotic early cell death. Consequently, in those probes, after PEFs exposure, even if the apoptosis pathway was activated, there were fewer cells left to enter the apoptosis^27^.

Some other studies indeed suggest that the main cell death mechanism induced by standalone nanosecond PEFs is apoptosis. Several phenomena associated with apoptosis were observed after the application of nsPEFs i.e. cytochrome c release^35^, caspase activation^35^ and DNA fragmentation^36^. In our study, the intensity of apoptosis was assessed by measuring the activation of caspase 3 and 7.

Ca^2+^ disturbances after nsPEFs were pointed to be one of the critical events leading to cell death^37^. NsPEFs trigger the increase of intracellular Ca^2+^ by its influx from the extracellular medium and efflux from the intracellular compartments^38^. Our study shows various Ca^2+^ transients evoked by ns bursts in different frequencies. Considering MHz pulses, the Ca^2+^ transients are similar to those obtained by a single longer pulse^24^. Bursts of kHz and Hz PEFs triggered variable responses. Ca^2+^ inflow has not occurred immediately after the delivery of pulses what suggests the involvement of different mechanisms. Dissynhronized response is likely caused by a delayed Ca^2+^ release from the intracellular compartments^38^.

Herein, we tried to find a correlation between cytoskeleton disruption and impaired cancer cell motility. Ns bursts affect the cytoskeleton as a result of permeabilization and subsequent cell swelling^39^. Presented confocal microscopy study showed more extensive cytoskeleton disruption after permeabilization with Ca^2+^. The observation has not been represented in the motility test. Exposure to PEFs, independent of extracellular Ca^2+^ concentration, affected the motility of cells. A small and insignificant difference between various therapy protocols could emerge from minor inequalities of viable cells seeded inside silicon inserts. Motility is a complex process, and apparently, changes in cytomechanics can not be entirely explained by actin and zyxin disruption.

Our study presented differences between high and low-frequency PEFs. High-frequency bursts introduce parametric flexibility. The extent of electroporation can be controlled solely by the frequency without altering the pulse energy, i.e., 1 kHz same parameters nsPEF will deliver lower electroporation than MHz. Moreover, some features of MHz compression of nsPEFs seem to be distinct from PEFs in other frequencies. Viability assays showed a more potent cytotoxic effect of MHz PEFs when applied in higher numbers and with extracellular Ca^2+^, over lower frequency pulses. Another interesting distinction was presented by Ca^2+^ uptake analysis. Previously it was speculated that the observed effects in MHz range are due to the accumulation of transmembrane potential and slow relaxation^22^. There are no data contradicting this hypothesis; thus, plasma membrane charge and discharge dynamics are considered primary mechanisms.

The possible effect of MHz nsPEFs on intracellular biomembranes is not yet understood. In theoretical analysis of MHz pulses, Sözer et al*.* (2021) denied charge accumulation and pointed at possible oscillations of the potential of intracellular membranes after MHz PEFs exposure^40^. It requires further experimental analysis to settle the question if MHz pulses have the potential of inducing the intracellular effects similar to nsPEFs in lower frequencies.

One of the rationales of applying short PEFs for the focal therapy of PCa is the possibility of ablations without thermal damage. However, the current flow results in the temperature rise due to Joule heating. In vivo nsPEFs showed rather a low capability of inducing the heating of tissue^41^. In our study, we applied ns pulses in a high-frequency range in a low conductivity medium. Considering the short duration (200 ns) and relatively low amplitude of pulses, the total energy of the highest intensity burst (1000 pulses) was low (< 0.25 J). Nevertheless, if the number of pulses and the amplitude is increased in the future, the induction of Joule heating should be considered. Other studies indicated an increase of the frequency from 1 Hz to 1 kHz showed the temperature rise of 3 °C of the electroporated tissue^42^. Another multi-parameter analysis of temperature rise after high-frequency electroporation shows that MHz compression of nanosecond pulses indeed has the potential of causing thermal damage due to Joule heating. The simulation performed on the electrical properties of liver tissue presented that burst of 100, 1 MHz pulses when the higher voltage is applied are likely to increase the tissue temperature by 8 °C^43^. In the in vitro study of Pakhomov et al., the calculated temperature rise after MHz PEFs did not exceed 6 °C^24^. It implies that Joule heating is specific to the case, but it is easy to control if the input energy is well defined. In our study, we can entirely exclude the thermal effect contributed to the observed cell death. However, if high conductivity (> 1 S/m) buffer is used in the future, the thermal effect should be reconsidered.

In this study, we proved the in vitro effectiveness of high-frequency nsPEFs for PCa treatment. Prospectively, ns pulses might provide additional advantages over μsPEFs, which are already applied for irreversible electroporation of PCa. High-frequency nsPEF allows mitigation of impedance changes. Therefore, a more uniform exposure of the tumor can be ensured. The inhomogeneity of various layers and structures is less a concern^44,45^. Bursts of ns pulses allow better control of the total treatment energy than protocols involving single pulses or low amplitude pulses. The ablation can be controlled in a more flexible and precise way if the number of pulses is high. Moreover, high-frequency nsPEFs are characterized by their low excitatory efficacy compared to longer pulses. With ns bursts, the permeabilization threshold for cancer cells can be exceeded, and simultaneously, the excitation threshold for surrounding neurons and muscle cells will not be reached^46^. Accordingly, the electroporation of cancer cells is possible without unwanted neuronal or muscular excitation and damage. That gives ns bursts an advantage namely, it excludes the need for complete muscle relaxation and general anesthesia during focal ablation of PCa.

## Materials and methods

### Cell culture

The androgen-independent prostate cancer cell line DU 145 and androgen-dependent prostate cancer cell line LNCaP were obtained from American Type Culture Collection and propagated as recommended by the supplier. Both media (RPMI 1640 and EMEM Sigma-Aldrich, St. Louis, MO, USA) were supplemented with 10% fetal bovine serum (FBS, Sigma-Aldrich) and 1% antibiotics (penicillin/streptomycin; Sigma-Aldrich). Cells were kept under standard culture conditions at 37 °C in a humidified atmosphere containing 5% CO_2_.

### Preparation of drug

Calcium solutions were prepared from the stock solution of calcium chloride (67 mg/ml, calcium chloratum WZF, Polfa Warszawa S.A., Poland). The required concentrations were achieved with a dilution of stock in EP buffer to 5 mM concentration.

### Permeabilization of cells

50 μl of cells in the concentration of 2 × 10^6^/ml, suspended in HEPES buffer (10 mM HEPES (Sigma-Aldrich), 250 mM sucrose (Chempur), and 1 mM MgCl_2_ (Sigma-Aldrich) in milliQ water) with or without CaCl_2_, were placed in 1 mm cuvettes with aluminium electrodes (BTX, Syngen Biotech, Poland). The square wave electroporator (100 ns–1 ms) developed in the Institute of High Magnetic Fields (VGTU, Vilnius, Lithuania) was used to deliver electric pulses^25^. Cells were exposed to 25 pulses of 200 ns, 7000 or 1000 V/cm and different frequencies ranging from 1 Hz to 1 MHz. Pulse shapes and amplitudes were controlled with an MDO3052 oscilloscope (Tektronix, Beaverton, OR). Subsequently, the cells were left incubating for 20 min and then suspended in a culture medium and placed in 96 or 6 well plates.

### Cell viability and caspase 3/7 activity assays

After permeabilization, the viability of cells was measured in 24 h with MTT assay^26^. We utilized a Caspase-Glo 3/7 Assay from Promega (Madison, WI,) to assess the caspase 3/7 activity in 8 h^27^. The luminescence and absorbance were measured with the multiplate reader GloMax (Promega, Madison, WI). The experiment was performed in triplicate.

### Cell permeability quantification assay

The cells were permeabilized according to the description of permeabilization of cell suspension. However, before PEFs delivery, the green-fluorescent YO-PRO-1 stain (Y3603, Thermo Fisher Scientific, Waltham, MA) in the concentration of 1 μl/ml was added to EP buffer. YO-PRO-1 cellular uptake reflects the degree of plasma membrane’s permeabilization^38^. After permeabilization cells were incubated for 3 min. Green Fluorescent intensities were detected on Cube 6 flow cytometer (Sysmex, Germany). The fluorescence of YO-PRO-1 was excited with 488 nm wavelength and measured with the FL-1- detector (525/50). The results were expressed as the percentage of permeabilized cells. The experiment was performed in triplicate.

### Calcium uptake evaluation

Cells were placed in a well of 6 wells plate. After 24 h the medium was removed, cells were stained with 4 μM Fluo-8 (ab142773, Abcam, UK) diluted in PBS and left incubating for 20 min. Subsequently, the buffer was replaced with HEPES or with HEPES with calcium chloride at a 5 mM concentration. The plate was placed on the microscope stage with the electrode touching the surface of the well. Cells visible in the objective were directly placed between two needles of the electrode BTX533 (BTX, Syngen Biotech, Poland). Subsequently, the cells were subjected to 200 ns PEFs with field intensity reaching around 5 kV/cm. The increase in cell fluorescence during the permeabilization was observed with the fluorescent microscope Olympus IX53 (Olympus, Japan). The changes in cell fluorescence were evaluated with ImageJ software (Version:2.1.0/1.53C; https://imagej.net/Fiji)^28^. The experiment was performed in triplicate.

### Qualitative evaluation of the cytoskeletal organization

The cells were permeabilized according to the protocol described above. Subsequently, the cells were seeded on glass coverslips placed and stained with primary zyxin antibody (MAB6977, RD systems, Minneapolis, MN) at 3 µg/ml and a mixture of Alexa Fluor 488 dye (Ex. 490 nm, Em. 525 nm; 2 μg/ml, A11029, Thermo Fisher Scientific Waltham, MA) and Alexa Fluor 546 Phalloidin (Ex. 556 nm, Em. 570 nm; 2 μg/ml, A22283, Thermo Fisher Scientific, Waltham, MA) according to the protocol^29^. Eventually, cells were mounted with DAPI Mounting Medium (ab104139, Abcam, UK). The samples were analyzed with a confocal laser scanning microscope using laser wavelengths: 405 nm, 490 nm, and 556 nm; 60 × oil immersion objective lens with 1.35 NA (Olympus FluoViewer 1000, Japan).

### Wound healing assay

After permeabilization, cells were seeded inside the silicone inserts (Ibidi, Germany) for 24 h to form a monolayer. Subsequently, the inserts were removed. The cells were scrutinized until the colonies of the control group connected. Note that PEFs with calcium as well as standalone MHz nsPEFs resulted in decreased viability of cell compared to control. Consequently, the proportionally higher number of cells that underwent the therapy was placed in the silicone insert for those samples. The changes in wound width were evaluated with ImageJ software (Version:2.1.0/1.53C; https://imagej.net/Fiji)^28^.
